# Early experience, structural dissociation, and emotional dysregulation in borderline personality disorder: the role of insecure and disorganized attachment

**DOI:** 10.1186/2051-6673-1-15

**Published:** 2014-10-28

**Authors:** Dolores Mosquera, Anabel Gonzalez, Andrew M Leeds

**Affiliations:** Instituto para el Tratamiento del Trauma y los Trastornos de Personalidad (INTRA-TP), A Coruña, Spain; Tu Clinica (Grupo Assistens), Universitary Hospital of A Coruña, A Coruña, Spain; Sonoma Psychotherapy Training Institute, 1049 Fourth St., Suite G, Santa Rosa, CA 95404-4345 USA

**Keywords:** Attachment, Borderline personality, Structural dissociation, Emotional regulation

## Abstract

Persistent problems in emotional regulation and interpersonal relationships in borderline patients can be understood as developing from difficulties in early dyadic regulation with primary caregivers. Early attachment patterns are a relevant causal factor in the development of Borderline Personality Disorder (BPD).

Links between attachment issues, early history of neglect, and traumatic experiences, and symptoms observed in patients with BPD as per the DSM-5 classification (American Psychiatric Association: Diagnostic and statistical manual of mental disorders: DSM-5 (Fifth ed.). Washington, D.C; (2013)) are described in this article, while delineating possible pathways from attachment disruptions to the specific symptomatology of these patients. The theory of structural dissociation of the personality (TSDP) provides an essential framework for understanding the processes that may lead from insecure early attachment to the development and maintenance of BPD symptoms.

Dyadic parent–child interactions and subsequent modulation of emotion in the child and future adult are considered closely related, but other factors in the development of BPD, such as genetic predisposition and traumatic experiences, should also be considered in conceptualizing and organizing clinical approaches based on a view of BPD as a heterogeneous disorder.

## Introduction

Borderline personality disorder (BPD) is characterized by many difficulties, including severe and persistent problems in emotional regulation and interpersonal relationships [1]. Borderline features are related to increased vulnerability to co-occurring moods, anxiety and eating disorders, and substance or alcohol abuse. In patients with BPD, core problems associated with impulse control and self-regulation tend to create other difficulties, such as angry outbursts, impulsive and self-mutilating behavior, fear of loneliness, identity disturbance, and a profound sense of emptiness. All these factors are interrelated and feed off each other [2].

Several authors describe problems of early attachment as a first order causal factor for the development of BPD [3–5]. While dyadic parent–child interactions and subsequent modulation of emotion in the child and future adult are closely related, other factors in the development of BPD, such as genetic predisposition and traumatic experiences, should also be considered. The theory of structural dissociation of the personality [6] provides an essential framework for understanding the processes that lead from insecure and disorganized early attachment to the development and maintenance of BPD symptoms. Consideration of all these factors conveys a view of BPD as a heterogeneous disorder with three general typologies.

In this paper, we examine the links between attachment issues, early history of neglect and traumatic attachment experiences, structural dissociation, and symptoms observed in BPD patients as per the DSM-5 classification [1]. The main objective of this article is to delineate the pathways from attachment disruptions to the specific symptomatology presented by these patients.

### An overview of the types of attachment

Bowlby [7] defines attachment behavior as an instinctive tendency, shown by humans and other higher species, to seek safety in proximity to a specific individual perceived as protective in situations in which fear or other feelings associated with perception of vulnerability are triggered.

Several authors have developed different models of attachment which describe categories and classifications characteristic of the infant-caregiver relationship, the current adult romantic attachment, and the adults’ retrospective description of early attachment [8–15].

Table 1 summarizes different attachment categories and attempts to reflect correspondences between subtypes included in the Adult Attachment Inventory (AAI); [16]; the most widely used instrument to measure adult descriptions of early attachment), the Strange Situation ([8, 9, 15]; an experimental situation exploring child-caregiver behaviors), and adult patterns of attachment in both romantic and peer relationships [10].

**Table 1:** **Different types of attachment in adults and children with different instruments**

AAI	Strange Situation	Adult romantic and peer relationships
**George et al. [** [16]**]**	**[** [8]**,** [9]**,** [15]**]**	**Bartholomew and Horowitz [** [10]**]**
**Secure/autonomous:** The person speaks coherently and interactively with the interviewer about life experiences, whether favorable or unfavorable. Questions are answered with sufficient, but not excessive, elaboration and provide a coherent narrative that may even include traumatic issues.	**Secure:** The infant seeks physical contact, proximity, and interaction. If upset by the separation, the infant is readily soothed by parents, and then returns to exploration and play.	**Secure:** “It is relatively easy for me to become emotionally close to others. I am comfortable depending on others and having others depend on me. I don't worry about being alone or having others not accept me.”
**Dismissing:** The person minimizes the discussion or importance of attachment-related experiences. Responses are typically internally inconsistent, and often excessively short. Relationships with parents are usually described as highly favorable, but without supporting evidence, or when it is given, tends to contradict the global evaluation.	**Insecure-avoidant:** These infants show little apparent separation anxiety while actually in a state of high physiological distress, avoid and ignore parents on reunion, remain occupied with toys, and may ignore parents’ efforts to communicate.	**Dismissing:** “I am comfortable without close emotional relationships. It is very important for me to feel independent and self-sufficient, and I prefer not to depend on others or have others depend on me.”
**Preoccupied:** The memories aroused by a question seem to draw the subject's attention and guide the subject's speech. This can result in lengthy, angry recounting of childhood interactions with parents, which may inappropriately move into discussions of a present relationship. The speaker may also digress to remote topics, use vague language, and describe a parent negatively and positively in the same sentence.	**Insecure-resistant.** These infants alternate between appearing very independent and ignoring mother and becoming anxious and trying to find her. Upon reunion, they cling and cry, but also look away and struggle, and parents are not able to sooth their distress.	**Preoccupied:** “I want to be completely emotionally intimate with others, but I often find that others are reluctant to get as close as I would like. I am uncomfortable without close relationships, but I sometimes worry that others don't value me as much as I value them.”
		Fearful: “I am somewhat uncomfortable getting close to others. I want emotionally close relationships, but I find it difficult to trust others completely, or to depend on them. I sometimes worry that I will be hurt if I allow myself to become too close to others.”
**Unresolved or disorganized:** Frequently demonstrates substantial lapses in reasoning or discourse. The respondent may express childlike beliefs or lapse into prolonged silence or eulogistic speech.	**Disorganized:** Infants cry for parents at the door and then run away when door opens, approaching parent with head down. Behavioral strategies seem to be collapsed. They may seem to freeze, display a vacant gaze, or engage in stereotyped behavior.	[Note: there is no corresponding category in the adult romantic and peer attachment relationship self-report literature for either the AAI disorganized or Strange Situation unresolved attachment groups.]

Note: As per Fraley and Shaver ([17], p. 1200): “The avoidant pattern in the three-category model [13] is represented by two patterns in Bartholomew's model: fearful-avoidance and dismissing-avoidance. Both of these patterns involve high scores on avoidance but differ on anxiety. Fearful-avoidance is a combination of high avoidance and high anxiety; dismissing-avoidance is a combination of high avoidance and low anxiety”.

### Theory of structural dissociation of the personality

In [18] developed a theory linking knowledge of psychotraumatology and neurobiology to classical theories on dissociation. Their model was called Theory of Structural Dissociation of the Personality (TSDP). This theory provides a useful theoretical framework for understanding BPD [19] and will be described in this article. Aside from consistent clinical evidence, studies are emerging that support the basic principles of the Theory of Structural Dissociation (e.g., [20, 21]; see [22], for a brief review).

TSDP provides a rich conceptualization of post-traumatic clinical pictures. The word dissociation is used to describe a mechanism involved in a fundamental division within the personality, which the authors believe is at the basis of all post-traumatic disorders. Post-traumatic clinical issues are distributed within a psychopathological spectrum ranging from acute trauma and PTSD on one end, to dissociative identity disorder on the other end, the most severe post-traumatic clinical profiles. In the middle of the spectrum we find borderline personality disorder.

Inspired by Allport [23] and Janet [24], Van der Hart et al. [6] define personality as the dynamic organization of those biopsychosocial systems within the individual that determine his or her characteristic mental and behavioral actions. Evolutionary prepared psychobiological action systems play a major role [6, 25, 26] in TSDP. One major set of action systems is oriented toward defense [27], escaping from and avoiding physical and associated psychological threat, and includes subsystems such as flight, freeze, fight, and total submission [28]. Other action systems are related to functions of daily life [26] and involve approaching attractive stimuli, energy regulation, attachment and care-taking, exploration, social engagement, play, and sexuality/reproduction [25].

TSDP thus postulates that in trauma - not only in criterion A trauma events, but also in what could be called attachment trauma - the personality may become divided among two or more such dissociative subsystems or parts [6]. Each part is primarily mediated by particular action (sub) systems and has its own first-person perspective. These dissociative parts, also known as dissociated self-states, are dysfunctionally stable (rigid) in their functions and actions, and overly separated from one another. One prototypical personality subsystem is metaphorically called the Emotional Part of the Personality (EP: [6, 29]). EPs are mediated by mammalian action systems of defense and attachment cry. As EPs, patients are fixated in reenactments of traumatic experiences. These reenactments include action tendencies of defense against perceived or actual threat to the integrity of the body or to life itself, as well as action tendencies regarding the need for attachment and the fear of attachment loss [30]. EPs are mediated by the innate action system of defense against threat that may be guided in particular by one of its subsystems: fight, flight, freeze, collapse, total submission, hypervigilance, wound care, and restorative states.

The other prototype personality subsystem is called the Apparently Normal Part of the Personality (ANP; [6, 29]). As ANP, the survivor experiences EP and at least some of EP’s actions and contents as ego-dystonic. As ANP, the patient is fixated in avoidance of traumatic memories and often of inner experience in general. Mediated by action systems for functioning in daily life, ANP focuses on the functions of these systems and, in this context, commonly seeks the approval of caretakers to gain acceptance, protection, and love. To the degree that such attachment-related goals are fulfilled, the painful result is that ANP’s appeasement and apparent normality are reinforced, not the survivor’s authenticity. ANP’s normality is only apparent, and manifests in negative symptoms of detachment, numbing, and partial or, in some cases, complete amnesia for the traumatic experience. As ANP, EP’s traumatic memories are experienced as ego-dystonic and intrusive symptoms such as voices, disowned thoughts, feelings or sensations, or acts that do not belong to their own sense of self or first person perspective (not-me experiences).

Another important concept is that of dissociative phobias [6], which maintain divisions within the overall personality. One example of these would be the phobia of traumatic memories. Patients may be phobic of mental contents (feeling, thinking about, “looking inside”) or may reject or disown specific parts of their personality. A strong conceptual grasp of these issues is crucial in guiding the therapeutic process.

According to TSDP, the structural dissociation of the personality will be more complex the greater the intensity, frequency, and duration of the traumatization and the earlier it started in life. BPD is usually related to severe and early traumatization and presents high levels of personality fragmentation, which is categorized in TSDP as secondary or tertiary structural dissociation [19].

Disorganized/disoriented attachment style [31–33], characteristic of dissociative disorders and a subgroup of borderline patients, can be understood from TSDP as an extreme alternation or competition between relational approach and defense against relational threat. Preoccupied and dismissing subtypes of insecure attachment can also be associated with BPD. These insecure attachment subtypes may also generate an alternation among non-integrated aspects of the personality, but in these cases, parts of the personality are generally less developed and structured than in disorganized attachment.

## Review

### Attachment theory and borderline personality disorder

Several authors have turned to Bowlby’s ideas to explain borderline pathology [3, 34]. Gunderson [35] proposes that intolerance of being alone is at the core of borderline pathology and that incapacity for calling on a “calming introjection” is the consequence of early attachment failures. He describes typical patterns of borderline dysfunction in relation to the exaggerated reactions of a child with insecure attachment – for example: holding on to other people, fear due to dependency needs, terror of abandonment, and constant monitoring of the caregiver‘s proximity. The need to check the closeness of others and the tendency to establish contact through demands for attention and requests for help seems to be related to preoccupied attachment.

Crittenden [36] pointed out the profound *ambivalence* and *fear of intimate relationships* in people with BPD. Lyons-Ruth and Jacobovitz [37] focused on the disorganization of the attachment system during childhood as the predisposing factor for a later borderline pathology. These authors identified that, as opposed to a secure pattern of attachment, either a disorganized or an insecure pattern predispose to behavioral problems.

Fonagy [38] and Fonagy et al. [39] stressed the importance of attachment in the development of symbolic function and how both insecure and disorganized attachment can lead to vulnerability. All these theoretical approaches, and others, predict that attachment representations in individuals with borderline pathology will be insecure or disorganized [34].

Paris [40] presents a biopsychosocial model in which he tries to explain how personality disorders are developed, in particular borderline personality disorder. He suggests that there are cumulative and interactive risk factors (biological, psychological, and social). He states that each child’s temperament may predispose to certain difficulties, but that temperament coupled with loss, trauma, or neglect experiences can make those traits become pathological. As an example, he explains that the majority of shy children (temperament) overcome shyness as they grow up, but if the family does not give them the necessary support, introversion can become accentuated (trait) and pathological (disorder). Shyness can drive the child to establish social contacts characterized by anxiety, withdrawal, and abnormal attachment patterns. If this continues over time, it becomes more complicated and ultimately the behaviors will fit the diagnostic criteria for dependent and avoidant personality disorder. From our point of view, these criteria are also manifested in people with BPD, both in their relationships and in their coping skills. Paris [40] pointed out that some people who develop BPD start life with temperamental characteristics compatible with normalcy (for example, a child who is less reflective and tends more toward action) and, perhaps, adequate psychosocial support could have prevented the development of a personality disorder. Paris [40] noted that parents of future adults with BPD may themselves have personality disorders, may be insensitive to the needs of their children, or may fail to provide an appropriate supportive environment. Positive experiences with secure attachment figures are one of the protective factors that have the most weight, but may or may not compensate sufficiently in those cases in which biological characteristics are prominent, which will be discussed later in this article.

Allen [41] proposes what he calls parental role confusion. He describes how some parents of people with BPD may be both obsessively focused on their children and simultaneously show anger at their children’s behavior (due to their limited capacities for affect tolerance and self-regulation), thereby creating one of the circumstances that can generate insecure preoccupied attachment. One way to understand this contradictory behavior in parents of people who develop BPD is to conceptualize it as a reaction to an intrapsychic conflict over the parenting role, a conflict generated and reinforced by the parents’ experiences in their own families of origin. Their ambivalence about being a parent would be the core theme of the relationship conflict Luborsky and Crits-Christoph [42]. They believe their duty is to sacrifice everything for their children, but at the same time, they feel overwhelmed by this responsibility and resent the sacrifice they have to make. When biologically predisposing factors are very prominent in the child, parents may become confused, frustrated, and angry when the extraordinary efforts they make fail to make a lasting impact on the maturational processes of their child. In some cases, feelings of failure as parents might emerge, especially when they receive contradictory information from professionals.

### Research on attachment and personality disorders, in particular borderline personality disorder

A number of studies link childhood attachment with the development of adult personality disorders and establish that insecure attachment is a relevant risk factor for the development of psychopathology.

Bakermans-Kranenburg and Van IJzendoorn [43] report that the normative attachment pattern with mothers in the general population is: 58% secure attachment, 23% dismissive, 19% preoccupied, and an additional 18% classified as unresolved attachment. In their extensive review of studies that used the Adult Attachment Interview (AAI) in the last 25 years, subjects in clinical samples had more insecure and unresolved-disorganized attachment than in the non-clinical groups.

Fonagy et al. [44] found that 92% of patients with BPD presented with insecure attachment (assessed through the AAI), especially preoccupied and unresolved-disorganized types. In a study of women with BPD, West et al. [45] mainly found early attachment relationships of the insecure preoccupied type. Patrick et al. [46] found an 83% of preoccupied attachment in a group of 12 patients with BPD. Barone et al. [47] found high rates (81% overall and as high as 97% in certain BPD diagnostic subgroups) of insecure and unresolved-disorganized attachment as assessed by the AAI in a large sample (N = 140) of BPD patients. The distribution of dismissing, preoccupied, and unresolved-disorganized classifications varied based on subgroups of internalizing or externalizing co-occurring Axis I disorders. While controlling for the influence of gender, childhood traumatic experiences, and the presence of an Axis I mental disorder, Nickell et al. [48] found that early insecure attachment is a significant predictor for BPD. Ling and Qian [49], in a sample of students, found a correlation between personality test scores and avoidance and anxiety in intimate relationships.

### Differential effects of insecure-preoccupied, insecure-dismissing, or unresolved-disorganized attachment in adults with borderline personality disorder

Bateman and Fonagy [3] propose that due to parental neglect and abuse (physical and psychological), people with BPD have an inadequate capacity to represent mental states: to recognize that their own reactions and those of others are motivated by thoughts, feelings, ideas, and hopes. The caregiver’s sensitivity to the child’s mental state is strongly related to secure attachment and the development of the child’s capacity to mentalize: to represent the behavior of self and others in relation to underlying mental states [50–54]. Nevertheless, Bateman and Fonagy [3] think that the descriptions of insecure attachment from childhood or adulthood provide an inadequate clinical explanation for several reasons: preoccupied attachment is very common and patterns of preoccupied attachment in infancy correspond to relatively stable adult strategies [55]. However, the hallmark of attachment disorders in borderline individuals is lack of stability [56]. This lack of stability can be understood from the framework of TSDP as alternating between different dissociative parts of the personality.

The alternation between one response and another seen in preoccupied attachment, the deactivation of affective states seen in dismissing insecure attachment, or the basic contradictions inherent to disorganized attachment lead to lack of integration. This relates to BPD’s diagnostic criterion 3 [1]: Identity Disturbance: markedly and persistently unstable self-image or sense of self. The challenge faced by some of these children is how to integrate a parent who sometimes becomes frightened when they cry or becomes upset when they get angry with a parent who at other times is available and loving. In other children with dismissing insecure attachment, the challenge can be the inability to draw on any parental response in the face of dysregulated affective states. These insurmountable challenges are associated with unregulated mental states that will resurface in the future every time individuals are triggered into affective states of sadness, fear, anger, or undefined discomfort, rendering them unable to modulate those emotions. This is related to other BPD criteria [1]: Affective instability due to a marked reactivity of mood (e.g.: intense episodic dysphoria, irritability or anxiety usually lasting a few hours and rarely a few days – Criterion 6) and inappropriate, intense anger or difficulty controlling anger (e.g.: frequent displays of temper, constant anger, recurrent physical fights – Criterion 8).

Incompatible and alternating mental states are considered in TSDP [6] as dissociative parts of the personality [19]. TSDP describes not only severe dissociative cases of Dissociative Identity Disorder (DID) or Dissociative Disorders Not Otherwise Specified (DDNOS), but also offers a model encompassing the full spectrum of trauma-based disorders, including chronic situations of dysfunctional attachment. Patients with structural dissociation of the personality do not have an integrated sense of self, but alternate between different mental states (parts of the personality) containing different emotions, different coping strategies, and different concepts of self and relationships.

Patients with BPD with a dismissing-insecure attachment may remain persistently disengaged from any emotional state that threatens the rigidly established persona they have created. These individuals cannot identify, manage, or regulate their emotions, because nobody helped them do so during childhood. When strong emotions appear, they may try to control them (resembling a more obsessive structure of the personality), but when this control fails, uncontrolled behaviors may lead to a borderline clinical picture, since they lack the skills to regulate or modulate those reactions.

When primary caregivers foster a preoccupied attachment style, we find adult patients who are unable to manage their anxiety: When they feel anxiety, or anger, or sadness, they quickly and automatically become overwhelmed. Preoccupied caregivers will have difficulties regulating emotional states in the child, and may even intensify them. Lacking healthy emotional regulation skills, substitute behaviors such as breaking things, driving recklessly, and hitting, frequent in BPD, may appear and complicate the clinical picture. When criticism, rejection, contempt, or hostility are also present in the interaction caregiver-child, these feelings could be unrecognized by the adult, but function as powerful triggers for impulsive, not conscious, and dysfunctional behaviors of different types. For example, a person who cannot recognize affective states of sadness or humiliation after a difficult social interaction will still experience intense physiological dysregulation and may turn to a compulsive ritual (work or solitaire) or even alcohol abuse in an attempt to manage the unmanageable. The most rejected parts or emotions are experienced as ego-dystonic, representing dissociative parts of the personality [6].

Disorganized early attachment generates vulnerability to extreme dissociation of the personality when combined with persistent childhood neglect and trauma. The caregiver is at the same time the source of protection and the source of danger [30, 33]. Attachment needs are conjoined with fear and defensive responses. This is an insurmountable biological paradox that can be addressed only by maintaining divisions within personality subsystems. Over time, in disorganized attachment with persistent childhood neglect and trauma, parts of the personality become more structured, containing aspects of reality and relationships that cannot be integrated. Quite autonomous mental structures can develop. Auditory hallucinations in BPD can be an example of dissociative parts of the personality. These voices are often mental phenomena arising from completely dissociated mental states. For example, a child growing up in an environment of physical abuse by a primary caregiver has a complex reaction to feeling anger. This emotion must be abolished, both as a means of protection from further attacks, and because it is identified with the abuser. Everything about this emotion is removed from the mind in order to carry on with daily life, creating an emotional part of the personality (EP) to contain this disavowed defensive action subsystem, [6]. The apparently normal part of the personality (ANP) is left with the responsibility for carrying out everyday life while trying to avoid content and emotions related to traumatic experience, without the benefits that normally accompany a well-integrated capacity for anger, such as boundary maintenance and assertiveness. But that which is set aside (intolerable anger) has become contained in a mental subsystem that is totally or partially disconnected from the rest. What emerges from this defensive subsystem can be experienced by ANP as an intrusive symptom: Thoughts or emotions that do not seem to be their own, or auditory hallucinations, which are interpreted as transient stress-related paranoid ideation or severe dissociative symptoms ([1]; criterion 9). The memories, which were the original sources of this emotional state, may be isolated within this trauma-derived subsystem. From the outside, we see this as amnesia: ANP cannot remember or recognize this source because the memories are “stored” in a fully dissociated emotional part of the personality (EP). When these memories are triggered, the patient (from a different emotional state) feels anger, activating at the same time different types of self-referent cognitions and behaviors, a different mental perspective.

The images of the “good” parent are connected to the attachment system, which is innately conditioned to attach to the parent. The images of the frightening parent are linked to a defensive action system, mediated by fear and anger, which is activated to protect from danger. When individuals with BPD start a relationship, the attachment system becomes initially activated. They easily idealize a new attachment figure, as they did with the parent. They attach with the intense and overwhelming need for affection that they felt as children and was never fully (or not at all) met. The need for attachment is very intense, and we often see this intensity as disproportionate, labeling it as cries for attention. In reality, it is disproportionate regarding the current situation, but it is absolutely proportionate to the unmet needs from the original situation. Since abrupt changes in the other were always the norm, the individual is hyper-alert to possible negative expressions in the other, constantly checking for the slightest hint of rejection. Since these incompatible and dissociated subsystems were never integrated, they remain imminent in diverse action systems, while still operating at a more primitive level of development. In this way, we see patients who are apparently “childish” or show “regressive” behavior, representing parts of the personality that can be understood as “stuck in time”.

### Attachment and self-regulation capacities

Developmental neurobiology studies examining self-regulation capacities show that, to a significant degree, the individual’s resilience depends on early attachment experiences [57–62]. Longitudinal studies on attachment show the continuation of childhood attachment patterns into adolescence and adulthood [63, 64].

Siegel and Hartzell [65] define three basic aspects in understanding how attachment is generated and how bonds between parents and children are established: attunement, balance, and coherence. As an adult, this securely attached individual will be capable of self-regulating, connecting with others, and seeking and receiving help. All these aspects are severely affected in patients with BPD. Early attachment may influence internal emotion regulation (self-soothing and self-calming capacities) and the possibility of regulating themselves through dyadic regulation. They often oscillate between becoming dependent and seeking regulation from others, considering themselves unable to manage their emotional states, and having significant difficulties in social engagement.

Problems with self-regulation may be influenced by a constitutional predisposition, due to acquired genetic, epigenetic, or biological factors, and by the dyadic learning of regulation, based on continuous daily interactions with primary caregivers during childhood. Children learn to recognize their internal states when they have a mirror, an attuned caregiver, who reflects, explains, and responds to them [66]. If what this mirror shows is discordant with what the child is feeling, or if there is no reflection, the inner world will not evolve toward emotional self-regulation. Parents who are more focused on their own needs than on those of their children, either due to self-centeredness, high levels of discomfort, or health or life problems taking up their energy, do not provide their children with enough opportunities to learn a complete emotional vocabulary. So it is easy for these children to grow up developing a tendency to ignore their own needs and focus on those of others.

Different types of attachment with primary caregivers may influence self-regulation in different ways [67]. There are some differences between preoccupied or dismissive insecure attachment and disorganized attachment. In **preoccupied or dismissive insecure attachment** (organized), the behavioral strategy is more or less always the same: “Since I cannot predict what my caregiver will do, if I cling (crying, screaming, and kicking), at least I can get her to be present.” Or, “I give up, I only have myself” (dismissive subtype). In this sense, the behavioral strategy approach is more or less stable or organized. In TSDP terms, the behavioral approaching sequence in **disorganized attachment** represents the simultaneous activation of two action systems, which should not be activated in that way at such a time. This attachment is chaotic because the approach does not end up truly taking place.

In insecure attachment (preoccupied or dismissive), the pattern seen in adults will basically be dependent or avoidant and will be related to what has been called “the attachment-based subtype of BPD” [19]. In disorganized attachment, responses more likely will be shifting and unstable, not establishing a stable bond precisely because that bond is a powerful trigger of the defensive fight/flight reactions. This group is associated with dissociative symptoms and rigid personality divisions, and has been called “the dissociative subtype of BPD” (see below). However, between insecure (preoccupied or dismissive) attachment and disorganized attachment there is a gradient in which, in addition to the lack of accessibility and inconsistency of the caregiver, varying degrees of hostility or overt aggression exist.

In closing, we again note that this article is not an attempt at proposing that only neglect, trauma, and attachment issues contribute to the development of borderline pathology. Impulsivity probably is a temperamental (i.e. genetic) trait in many cases [68]. The relationship between BPD, ADHD, bipolar disorder, and schizophreniform disorder becomes evident when we see the longitudinal course of many of these cases. Genetic factors highlighted by some models (see for example, [69]) best describe a subgroup of borderline patients. In this article, however, we have tried to examine the features of borderline pathology from the perspective of attachment theories and TSDP in order to develop an understanding of how early experiences can contribute to the psychopathology of this personality disorder.

## Conclusions

Attachment issues alone neither can explain the complexity of BPD, nor should they be seen as the sole cause for the development of borderline personality disorder. They should be understood as one piece of the puzzle. A wide range of studies show that early attachment seems to be linked to the development of borderline pathology, but each attachment pattern may generate different problems in emotion regulation, leading to different sets of borderline features and heterogeneous subtypes of BPD. The theory of structural dissociation of the personality may give us a framework for understanding the pathways from early attachment patterns to adult psychopathology, which are presented in this article as a hypothetical model.

## References

[1] American Psychiatric Association (2013). Diagnostic and Statistical Manual of Mental Disorders: Dsm-5.

[2] Mosquera D (2010). Trastorno Límite de la Personalidad. Una aproximación conceptual a los criterios del DSM-IV-TR Revista Persona.

[3] Bateman A, Fonagy P (2004). Psychotherapy for Borderline Personality Disorder: Mentalization Based Treatment.

[4] Kernberg P, Weiner A, Bardenstein K (2000). Personality Disorders in Children and Adolescents.

[5] Mosquera D, Gonzalez A (2009). Disociación estructural y trastorno límite de la Personalidad. Trastornos de la Personalidad. I Jornadas gallegas.

[6] Van der Hart O, Nijenhuis E, Steele K (2006). The haunted self: structural dissociation and the treatment of chronic traumatization.

[7] Bowlby J (1988). A Secure Base: Parent–Child Attachment and Healthy Human Development.

[8] Ainsworth MDS, Blehar MC, Waters E, Wall S (1978). Patterns of Attachment: A Psychological Study of the Strange Situation.

[9] Ainsworth MD, Blehar MC, Waters E, Wall S (1978). Patterns of Attachment: Assessed in the Strange Situation and at Home.

[10] Bartholomew K, Horowitz LM (1991). Attachment styles among young adults: a test of a four-category model. J Pers Soc Psychol.

[11] Fraley RC, Spieker SJ (2003). Are infant attachment patterns continuously or categorically distributed? A taxometric analysis of strange situation behavior. Dev Psychol.

[12] Fraley RC, Waller NG, Brennan KA (2000). An item-response theory analysis of self-report measures of adult attachment. J Pers Soc Psychol.

[13] Hazan C, Shaver P (1987). Romantic love conceptualized as an attachment process. J Pers Soc Psychol.

[14] Hesse E, Cassidy J, Shaver PR (1999). The Adult Attachment Interview: Historical and Current Perspectives. Handbook of Attachment: Theory, Research and Clinical Applications.

[15] Main M, Solomon J, Brazelton, Yogman (1986). Discovery of a New, Insecure-disorganized/Disoriented Attachment Pattern. Affective Development in Infancy.

[16] George C, Kaplan N, Main N (1985). Adult Attachment Interview: Unpublished Manuscript.

[17] Fraley RC, Shaver PR (1998). Airport separations: a naturalistic study of adult attachment dynamics in separating couples. J Pers Soc Psychol.

[18] Van der Hart O, Nijenhuis E, Steele K, Brown D (2004). Trauma-related dissociation: conceptual clarity lost and found. Aust N Z J Psychiatry.

[19] Mosquera D, Gonzalez A, Van der Hart O (2011). Borderline personality disorder, childhood trauma and structural dissociation of the personality. Revista Persona.

[20] Reinders AATS, Nijenhuis ETS, Paans AMJ, Korf J, Willemsen ATM, den Boen JA (2003). One brain, two selves. Neuroimage.

[21] Reinders AATS, Nijenhuis ER, Quak J, Korf J, Haaksma J, Paans AM, den Boen JA (2006). Psychobiological characteristics of dissociative identity disorder: a symptom provocation study. Biol Psychiatry.

[22] Van der Hart O, Nijenhuis E, Solomon R (2010). Dissociation of the personality in complex trauma-related disorders and EMDR: theoretical considerations. J EMDR Pract Res.

[23] Allport GW (1981). Personality and Social Encounter.

[24] Janet P (1907). The Major Symptoms of Hysteria.

[25] Lang PJ (1995). The emotion probe: studies of motivation and attention. Am Psychol.

[26] Panksepp J (1998). Affective Neuroscience: The Foundations of Human and Animal Emotions.

[27] Fanselow MS, Lester LS, Bolles RC, Beecher MD (1988). A Functional Behavioristic Approach to Aversively Motivated Behavior: Predatory Imminence as a Determinant of the Topography of Defensive Behavior. Evolution and Learning.

[28] Porges SW (2003). The polyvagal theory: phylogenetic contributions to social behavior. Physiol Behav.

[29] Myers CS (1940). Shell Shock in France 1914–1918.

[30] Liotti G (1999). Understanding the dissociative processes: the contribution of attachment theory. Psychoanal Inq.

[31] Liotti G (1992). Disorganized-disoriented attachment in the etiology of dissociative disorders. Dissociation.

[32] Liotti G, Solomon J, George C (1999). Disorganized Attachment and Dissociative Psychopathology: the Contribution of Attachment Theory. Attachment Disorganization.

[33] Liotti G, Danquah AN, Berry K (2014). Disorganised Attachment in the Pathogenesis and the Psychotherapy of Borderline Personality Disorder. Attachment Theory in Adult Mental Health: A Guide to Clinical Practice.

[34] Fonagy P, Bateman A, Oldham JM, Skodal AE, Bender DS (2005). Attachment Theory and Mentalization-Oriented Model of Borderline Personality Disorder. The American Psychiatric Publishing Textbook of Personality Disorder.

[35] Gunderson JG (1996). The borderline patient’s intolerance of aloneness: insecure attachments and therapist availability. Am J Psychiatr.

[36] Crittenden PM (1997). Peering into the Black Box: An Exploratory Treatise on the Development of the Self in Young Children. Rochester Symposium on Developmental Psychopathology.

[37] Lyons-Ruth K, Jacobovitz D, Cassidy J, Shaver PR (1999). Attachment Disorganization: Unresolved Loss, Relational Violence and Lapses in Behavioral and Attentional Strategies. Handbook of Attachment Theory and Research.

[38] Fonagy P (2000). Attachment and borderline personality disorder. J Am Psychoanal Assoc.

[39] Fonagy P, Target M, Gergely G (2000). Attachment and borderline personality disorder: a theory and some evidence. Psychiatr Clin N Am.

[40] Paris J (1994). Borderline Personality Disorder. A Multidimensional Approach.

[41] Allen DM (2003). Psychotherapy with Borderline Patients. An Integrated Approach.

[42] Luborsky L, Crits-Christoph P (1990). Understanding transference: The Core Conflictual Relationship Theme Method.

[43] Bakermans-Kranenburg M, Van IJzendoorn M (2009). The first 10,000 adult attachment interviews: distributions of adult attachment representations in clinical and non-clinical groups. Attach Hum Dev.

[44] Fonagy P, Leigh T, Steele M, Steele H, Kennedy R, Mattoon G, Gerber A (1996). The relation of attachment status, psychiatric classification, and response of psychotherapy. J Consult Clin Psychol.

[45] West M, Keller A, Links P, Patrick J (1993). Borderline disorder and attachment pathology. Can J Psychiatr.

[46] Patrick M, Hobson RP, Castle D, Howard R (1994). Personality disorder and the mental representation of early social experience. Dev Psychopathol.

[47] Barone L, Fossati A, Guiducci V (2011). Attachment mental states and inferred pathways of development in borderline personality disorder: a study using the adult attachment interview. Attach Hum Dev.

[48] Nickell AD, Waudby CJ, Trull TJ (2002). Attachment, parental bonding, and borderline personality disorder features in young adults. J Personal Disord.

[49] Ling H, Qian M (2010). Relationships between attachment and personality disorder symptoms of Chinese college students. Soc Behav Persona.

[50] Fonagy P, Steele H, Steele M (1991). Maternal representations of attachment during pregnancy predict the organization of infant-mother attachment at one year of age. Child Dev.

[51] Fonagy P, Target M (1996). Playing with reality, I: theory of mind and the normal development of psychic reality. Int J Psychoanal.

[52] Meins E, Fernyhough C (1999). Linguistic acquisitional style and mentalising development: the role of maternal mind-mindedness. Cogn Dev.

[53] Meins E, Fernyhough C, Fradley E, Tuckey M (2001). Rethinking maternal sensitivity: mothers’ comments on infants’ mental processes predict security of attachment at 12 months. J Child Psychol Psychiatr Allied Discipline.

[54] Meins E, Russel J (1997). Security and symbolic play: the relation between security of attachment and executive capacity. Br J Dev Psychol.

[55] Main M, Kaplan M, Cassidy J (1985). Security in infancy, childhood and adulthood: a move to the level of representation. Monogr Soc Res Child Dev.

[56] Higgitt A, Fonagy P (1992). Psychotherapy in borderline and narcissistic personality disorder. Br J Psychiatry.

[57] Fonagy P, Gergely G, Jurist E, Target M (2002). Affect Regulation, Mentalization, and the Development of the Self.

[58] Schore A (2003). Affect Regulation and the Repair of the Self.

[59] Schore A (2003). Affect Dysregulation and the Repair of the Self.

[60] Siegel D (1999). The Developing Mind. Toward a Neurobiology of Interpersonal Experience.

[61] Teicher MH (2002). Scars that won’t heal: the neurobiology of child abuse. Sci Am.

[62] Teicher MH, Gold CA, Surrey J, Sweet C (1993). Early childhood abuse and limbic system ratings in adult psychiatric outpatients. J Neuropsychiatry Clin Neurosci.

[63] Carlson E, Sroufe LA, Cicchetti D, Cohen D (1995). The Contribution of Attachment Theory to Developmental Psychopathology. Developmental Processes and Psychopathology: Volume 1. Theoretical Perspectives and Methodological Approaches.

[64] Demos EV, Goldberg (1998). Affect and the Development of the Self: A New Frontier. Frontiers in Self Psychology.

[65] Siegel DJ, Hartzell M (2004). Parenting from the Inside Out.

[66] Siegel DJ (2001). The Developing Mind: How Relationships and the Brain Interact to Shape Who We Are.

[67] Mosquera D, Gonzalez A (2014). Trastorno límite de la personalidad y terapia EMDR.

[68] Kochanska G, Philibert RA, Barry RA (2009). Interplay of genes and early mother-child relationship in the development of self- regulation from toddler to preschool age. J Child Psychol Psychiatry.

[69] Zimmerman P, Mohr C, Spangler G (2009). Genetic and attachment influences on adolescents’ regulation of autonomy and aggressiveness. J Child Psychol Psychiatry.

